# Root-zone-specific sensitivity of K^+^-and Ca^2+^-permeable channels to H_2_O_2_ determines ion homeostasis in salinized diploid and hexaploid *Ipomoea trifida*

**DOI:** 10.1093/jxb/ery461

**Published:** 2019-01-25

**Authors:** Yang Liu, Yicheng Yu, Jianying Sun, Qinghe Cao, Zhonghou Tang, Meiyan Liu, Tao Xu, Daifu Ma, Zongyun Li, Jian Sun

**Affiliations:** 1 Jiangsu Key Laboratory of Phylogenomics and Comparative Genomics, School of Life Sciences, Jiangsu Normal University, Xuzhou, Jiangsu, China; 2 Sweet Potato Research Institute (CAAS), Jiangsu Xuzhou Sweet Potato Research Institute, MOA Key Laboratory of Biology and Genetic Improvement of Sweet Potato, Xuzhou, Jiangsu, China

**Keywords:** Ca^2+^ transport, H_2_O_2_, K^+^/Na^+^ homeostasis, K^+^ retention, Na^+^ exclusion, polyploid, salinity stress

## Abstract

Polyploids generally possess superior K^+^/Na^+^ homeostasis under saline conditions compared with their diploid progenitors. In this study, we identified the physiological mechanisms involved in the ploidy-related mediation of K^+^/Na^+^ homeostasis in the roots of diploid (2*x*) and hexaploid (6*x*; autohexaploid) *Ipomoea trifida*, which is the closest relative of cultivated sweet potato. Results showed that 6*x I. trifida* retained more K^+^ and accumulated less Na^+^ in the root and leaf tissues under salt stress than 2*x I. trifida.* Compared with its 2*x* ancestor, 6*x I. trifida* efficiently prevents K^+^ efflux from the meristem root zone under salt stress through its plasma membrane (PM) K^+^-permeable channels, which have low sensitivity to H_2_O_2_. Moreover, 6*x I. trifida* efficiently excludes Na^+^ from the elongation and mature root zones under salt stress because of the high sensitivity of PM Ca^2+^-permeable channels to H_2_O_2_. Our results suggest the root-zone-specific sensitivity to H_2_O_2_ of PM K^+^- and Ca^2+^-permeable channels in the co-ordinated control of K^+^/Na^+^ homeostasis in salinized 2*x* and 6*x I. trifida*. This work provides new insights into the improved maintenance of K^+^/Na^+^ homeostasis of polyploids under salt stress.

## Teaser

Low sensitivity to H_2_O_2_ of PM K^+^-permeable channels in the meristem root zone and high sensitivity of PM Ca^2+^-permeable channels in the elongation and mature root zones are correlated with ploidy-dependent K^+^ retention and Na^+^ exclusion under salt stress.

## Introduction

Polyploidization or whole-genome duplication (WGD) is a pervasive biological phenomenon and the main driving force in plant evolution (Adams and Wendel, 2005; del Pozo and Ramirez-Parra, 2015). Most angiosperms have undergone at least one round of WGD during their evolution (Parisod *et al.*, 2010). Many important crop and horticultural plants, including wheat (allohexaploid), cotton (allotetraploid), oilseed rape (allotetraploid), sweet potato (autohexaploid), grape (tetraploid), and strawberry (octaploid) are polyploids, which can develop novel transcriptional, post-transcriptional, physiological, and morphological features that enhance their adaptability to environmental stresses relative to that of their diploid progenitors (Parisod *et al.*, 2010; del Pozo and Ramirez-Parra, 2015; Liu and Sun, 2017). For example, the autotetraploids *Arabidopsis thaliana* (del Pozo and Ramirez-Parra, 2014) and *Citrus limonia* (Allario *et al.*, 2013) are more tolerant to water deficiency than their diploid counterparts. An in-depth understanding of the physiological and molecular mechanisms underlying the differential stress adaptation of polyploids and their diploid progenitors is important to polyploid crop improvement (del Pozo and Ramirez-Parra, 2015).

Salinity stress reduces growth, impairs ion homeostasis, and disrupts metabolic processes in plants, and markedly decreases crop yield in irrigated lands (Shabala *et al.*, 2016*a*). Generally, retaining high K^+^ levels and accumulating low Na^+^ concentrations in the cytosol are essential to salinity stress tolerance in plants (Munns and Tester, 2008; Anschütz *et al.*, 2014; Deinlein *et al.*, 2014). The polyploid accessions of Arabidopsis and the allohexaploid wheat are more tolerant to salt stress and are better at mediating K^+^/Na^+^ homeostasis under saline conditions than their respective diploid progenitors (Chao *et al.*, 2013; Yang *et al.*, 2014). However, the reasons for this ploidy-determined differential K^+^/Na^+^ homeostasis regulation are still largely unknown.

Sweet potato, *Ipomoea batatas* (L.) Lam., is a hexaploid crop that is rich in nutrients and is ranked as the seventh most important food crop in the world and the fourth most significant food crop in China (Yang *et al.*, 2017). The wild relative species of sweet potato possess many desirable traits, such as abiotic and biotic resistance, thus contributing to the breeding and improvement of cultivated sweet potato (Isobe *et al.*, 2017; Liu, 2017). Recently, genome sequencing and nuclear/chloroplast phylogenies demonstrated that *Ipomoea trifida* (H.B.K.) G. Don. is the closest relative of modern cultivated sweet potato, although the existence of other extant species involved in the evolution of *I. batatas* remains controversial (Yang *et al.*, 2017; Muñoz-Rodríguez *et al.*, 2018). Diverse ploidy levels, ranging from diploid (2*n*=2*x*=30) to hexaploid (2*n*=6*x*=90) in *I. trifida*, have been found in nature (Hirakawa *et al.*, 2015). New evidence from restriction site-associated DNA sequencing revealed that cultivated sweet potato probably originated from hexaploid (6*x*) *I. trifida*, which evolved from diploid (2*x*) *I. trifida* (Feng *et al.*, 2018). Thus, *I. trifida* with different ploidy levels are ideal experiment materials for investigating the mechanisms involved in ploidy-determined K^+^/Na^+^ homeostasis.

The reduction in NaCl-induced K^+^ efflux from root tissues may help plants to achieve K^+^ homeostasis at the whole-plant level under saline conditions (Shabala and Pottosin, 2014). The magnitude of NaCl-induced K^+^ efflux is strongly correlated with cellular K^+^ retention and salt tolerance in a broad range of species, including barley (Chen *et al.*, 2005), wheat (Cuin *et al.*, 2008), poplar (Sun *et al.*, 2009*a*), sweet potato (Yu *et al.*, 2016), *Brassica* species (Chakraborty *et al.*, 2016), and halophytes (Bose *et al.*, 2015; Zeng *et al.*, 2018). The following mechanisms mediate K^+^ efflux from root tissues in salinized plants: (i) depolarization-activated outward-rectifying K^+^-permeable channels (DA-KORCs); (ii) weakly voltage-dependent non-selective cation channels (NSCCs); and (iii) reactive oxygen species (ROS)-activated K^+^-permeable channels (including KORCs and NSCCs) (Shabala *et al.*, 2016*b*). Thus, high plasma membrane (PM) H^+^-ATPase activity and low ROS accumulation are two important traits that contribute to the inactivation of DA-KORCs and ROS-activated K^+^-permeable channels, thereby decreasing the K^+^ efflux caused by high salinity (Anschütz *et al.*, 2014; Shabala and Pottosin, 2014; Shabala *et al.*, 2016*b*). Thus, in this study, we determined whether K^+^ efflux in the roots plays a major role in K^+^ homeostasis in polyploids under saline conditions.

PM Na^+^/H^+^ antiporter [salt overly sensitive 1 (SOS1)]-mediated Na^+^ exclusion is used by many plants to achieve cytosolic Na^+^ homeostasis under saline conditions (Sun *et al.*, 2009*b*; Cuin *et al.*, 2011; Maathuis, 2014). The activation of SOS1 is mediated by a Ca^2+^-dependent pathway, in which a salt-induced cytosolic Ca^2+^ increment is sensed by SOS3 (a myristoylated calcium-binding protein) and then activated by recruiting SOS2 (a serine/threonine protein kinase) to form the SOS3–SOS2 complex, which phosphorylates the C-terminus of SOS1 to activate Na^+^ extrusion activity (Qiu *et al.*, 2002; Zhu, 2016). PM NADPH oxidase-mediated ROS production under saline conditions is important for the regulation of Na^+^ homeostasis in different plant species (Zhang *et al.*, 2007; Chung *et al.*, 2008; Sun *et al.*, 2010; Ma *et al.*, 2012; Niu *et al.*, 2018). Several mechanisms were reported to explain the ROS-dependent mediation of Na^+^ homeostasis, including stabilizing *SOS1* mRNA (Chung *et al.*, 2008), stimulating PM H^+^-ATPase activity (Zhang *et al.*, 2007; Niu *et al.*, 2018), and activating PM Ca^2+^-permeable channels to reinforce [Ca^2+^]_cyt_ signaling (Sun *et al.*, 2010; Ma *et al.*, 2012). Therefore, we also determined whether Na^+^ extrusion, the Ca^2+^ transport system, and H_2_O_2_ production in the roots are involved in the efficient mediation of Na^+^ homeostasis in polyploids.

The root-zone- or cell-type-specific responses in roots are important to the salt tolerance of plants (Dinneny, 2010). Shabala *et al.* (2016*b*) used various electrophysiological techniques to demonstrate a cell-type-specific response of ion transport to salinity in barley roots. The root apex is highly sensitive to salinity compared with the mature region, and the K^+^ efflux caused by the weak PM H^+^-ATPase activity determines the K^+^ status of whole plants under saline conditions (Shabala *et al.*, 2016*b*). Numerous genes are expressed in a cell-type-specific manner, in terms of both longitudinal and radial root profiles (Dinneny *et al.*, 2008), and salt-regulated cell-type-specific transcriptional responses are essential to water transport modification, Casparian strip formation, and protein translation (Geng *et al.*, 2013). In addition, a root-zone-specific accumulation of metabolites in barley may have a potential role in the maintenance of root cell division and elongation under saline conditions (Shelden *et al.*, 2016). Therefore, we investigated whether the root-zone-specific pattern of ion transport contributes to the enhanced K^+^/Na^+^ homeostasis in polyploids.

In the present study, we compared the ion flux patterns (K^+^/H^+^/Ca^2+^/Na^+^) from various root zones in 6*x* and 2*x I. trifida* in response to NaCl or ROS stimuli using the non-invasive micro-test technology (NMT). Combined with pharmacological experiments, the results presented here demonstrated that the root-zone-specific sensitivity of PM K^+^- and Ca^2+^-permeable channels to H_2_O_2_ determines the differential capacity of K^+^/Na^+^ homeostasis regulation in salinized 2*x* and 6*x I. trifida*. Our results may provide insights into how autohexaploids provide improved K^+^/Na^+^ homeostasis under saline conditions in plants.

## Materials and methods

### Plant material and experimental condition

The seedlings of 2*x* and 6*x I. trifida* were obtained from the Key Laboratory of Biology and Genetic Improvement of Sweet Potato, Sweet Potato Research Institute, Xuzhou, Jiangsu, China. The ploidy level and the number of chromosomes in 2*x* (2*n*=2*x*=30) and 6*x* (2*n*=6*x*=90) *I. trifida* were confirmed via cytogenetic analysis (see Supplementary Fig. S1 at *JXB* online). Then, these seedlings were used for peeling stem apexes. The separated stem apexes were cultured on a regeneration medium [Murashige and Skoog (MS) medium supplied with 0.2 mg l^−1^ naphthaleneacetic acid (NAA) and 0.2 mg l^−1^ 6-benzylaminopurine (6-BA)] to obtain the regenerated virus-free plantlets. The virus-free plantlets were transferred to plastic pots containing peat moss and loamy soil at a ratio of 1:1 and placed inside a clean greenhouse for stem-cutting propagation. After enough seedlings were obtained, the shoots (with 3–5 mature leaves) of 2*x* and 6*x I. trifida* were cut and immersed in non-buffered quarter-strength Hoagland solution [containing 1.25 mM KNO_3_, 1 mM Ca(NO_3_)_2_, 1 mM MgSO_4_, 0.25 mM NH_4_NO_3_, 0.25 mM KH_2_PO_4_, 10 μM EDTA-Fe, 1.25 μM KI, 25 μM H_3_BO_3_, 25 μM MnSO_4_, 12.5 μM ZnSO_4_, 0.25 μM Na_2_MoO_4_, and 0.025 μM CuSO_4_, pH 5.7] to initiate adventitious root growth for 5 d and 10 d, respectively (adventitious root induction in 6*x* was faster than in 2*x I. trifida*). The cuttings were continuously aerated by passing air through the solution. The temperature in the greenhouse ranged from 20 °C to 25 °C with a photoperiod of 16 h, and with a photosynthetic photon flux density of 300 μmol m^–2^ s^–1^. Afterwards, uniform rooted seedlings (with similar shoots and root length; Supplementary Fig. S2) were selected for recording of the transient flux kinetics (NaCl and ROS stimuli) or subjected to 150 mM NaCl for 7 d. The required amount of NaCl was added to quarter-strength Hoagland nutrient solution at the beginning of treatment to reach 150 mM. At corresponding time points, the fine roots were collected for measurements of PM integrity, K^+^ and Na^+^ content (root and leaf tissues), steady-state root ion fluxes (K^+^ and Na^+^), intracellular Na^+^ accumulation, and H_2_O_2_ production.

### Determination of ploidy level

The root tips were pre-treated with 2 mM 8-hydroxyquinoline for 2 h at room temperature and fixed in ethanol–acetic acid. The procedure for chromosome preparation followed the protocol as described in Han *et al.* (2011). The images were captured by using an Olympus BX63 epifluorescence microscope after the chromosomes were counterstained by DAPI in a Vectashield antifade solution (Vector Laboratories).

### Determination of PM integrity

The PM integrity in root cells was checked by using propidium iodide (PI) staining as described in Sun *et al.* (2012). Root tips (3 cm) were collected from non-treated or NaCl-treated 2*x* and 6*x I. trifida* and were incubated in staining buffer containing 5 mM KCl/MES and 3 μg ml^−1^ PI (Life Technologies, Carlsbad, CA, USA) for 20 min. The samples were then washed in KCl/MES buffer for 5 min before imaging (elongation root zone) with an Olympus BX63 epifluorescence microscope.

### Determination of K and Na contents

After 7 d of 150 mM NaCl treatment, the seedlings were divided into two parts (the root and leaf), and the root tissues were washed in a culture dish containing deionized water three times for 2 min each. Thereafter, the fresh samples were dried in an oven at 70 °C to constant weight. The dried samples were weighed and pulverized, and were digested with concentrated H_2_O_2_ and HClO_4_ (7:1 v/v) in a microwave oven (Mars CEM 240/50) and subjected to inductively coupled plasma MS analysis (Agilent7500a, USA) to determine the concentrations of K and Na (Yu *et al.*, 2018).

### Visualization of H_2_O_2_ in different root zones

H_2_O_2_ was visualized in the different root zones by using a green ﬂuorescent probe, 2′,7′-dichlorodihydrofluorescein diacetate (H_2_DCF-DA; Sun *et al.*, 2010). After 5 d of NaCl treatment, root segments (3 cm) were collected and treated with 50 μM H_2_DCF-DA (Life Technologies) (prepared in a 5 mM KCl/MES buffer, pH 5.7 adjusted with HCl/KOH) for 10 min at room temperature in the dark. Thereafter, the H_2_DCF-DA-loaded root segments were washed several times with KCl/MES buffer. The DCF-dependent ﬂuorescence from meristem, elongation, and mature root zones was measured with an Olympus BX63 epifluorescence microscope. The root segments collected from the control seedlings were treated with H_2_DCF-DA as described above. Microscopic measurements were conducted after 30 min of NaCl (150 mM) application. H_2_O_2_ levels (in arbitrary unit) in specific regions (area of interest; AOI) were measured with an image processing software (Image-Pro Plus 6.0).

### Visualization of Na^+^ in mature root cells

The Na^+^-specific fluorescent dye, CoroNa-Green AM (Life Technologies) was used to visualize the Na^+^ accumulation in the root cells of 2*x* and 6*x I. trifida* (Sun *et al.*, 2012). After 5 d of NaCl treatment, the fine roots were collected and transferred to a fresh nutrient solution containing 150 mM NaCl, 20 μM CoroNa-Green AM, and 0.02% pluronic acid (Life Technologies) for 2 h. The roots were then washed 3–4 times for 1 min each with distilled water, and the intracellular Na^+^ fluorescence (mature root zone) was visualized under an Olympus BX63 epifluorescence microscope.

### Measurement of K^+^, H^+^, Ca^2+^, and Na^+^ fluxes

The net ﬂuxes of K^+^, H^+^, Ca^2+^, and Na^+^ were determined using a NMT system (NMT-100SIM-YG, YoungerUSA LLC, Amherst, MA, USA) as described in previous studies (Sun *et al*., 2009*a*, *b*; Yu *et al*., 2016, 2018). The construction of K^+^-, H^+^-, Ca^2+^-, and Na^+^-selective microelectrodes (2–4 μm tip diameter) followed standard procedures (Sun *et al*., 2009*a*, *b*; Yu *et al.*, 2016). The ion-selective microelectrodes for the target ions were calibrated before ﬂux measurements: (i) K^+^: 0.1, 0.5, and 1.0 mM KCl [background solution: 0.1 mM MgCl_2_, 0.1 mM CaCl_2_, and 150 mM NaCl, pH was adjusted to 5.7 with NaOH and HCl; 150 mM NaCl was replaced by 10 mM H_2_O_2_ or 1 mM hydroxyl radicals (OH·)-generating copper/ascorbate mixture (Cu/A) in the ROS experiments]; (ii) H^+^: pH 5.0, 6.0, and 7.0 (background solution: 0.1 mM MgCl_2_, 0.1 mM CaCl_2_, 0.5 mM KCl, 150 mM NaCl; pH was adjusted with NaOH and HCl); (iii) Ca^2+^: 0.1, 0.5 and 1.0 mM CaCl_2_ (background solution: 0.1 mM MgCl_2_, 0.5 mM KCl, 150 mM NaCl, pH was adjusted to 5.7 with NaOH and HCl; 150 mM NaCl was replaced by 10 mM H_2_O_2_ or 1 mM Cu/A in the ROS experiments); (iv) Na^+^: 0.1, 0.5, and 1.0 mM NaCl (background solution: 0.1 mM MgCl_2_, 0.1 mM CaCl_2_, and 0.5 mM KCl, pH was adjusted to 5.7 with KOH and HCl). Ion-selective microelectrodes with Nernstian slopes >52 mV per decade for K^+^, H^+^, and Na^+^ (26 mV per decade for Ca^2+^) were used. Ion ﬂux was calculated as described previously (Sun *et al*., 2009*a*, *b*; Yu *et al.*, 2016).

### Transient K^+^, H^+^, and Ca^2+^ flux measurements

Root segments with 3 cm apices were sampled from 2*x* and 6*x I. trifida* (control seedlings) for transient K^+^, H^+^, and Ca^2+^ flux measurements. The root segments were transferred to the measuring chamber containing 5 ml of fresh measuring solution (containing 0.1 mM NaCl, 0.1 mM MgCl_2_, 0.1 mM CaCl_2_, and 0.5 mM KCl at pH 5.7) for 30 min. The steady ﬂuxes of K^+^, H^+^, and Ca^2+^ were recorded from the meristem (500 μm from the tip), elongation (3 mm from the tip), and mature (15 mm from the tip) root zones for 5 min before salt and ROS treatment. Thereafter, salt and ROS treatment were performed by adding NaCl (final concentration of 150 mM), H_2_O_2_ (final concentration of 10 mM), and Cu/A (final concentration of 1 mM). All treatment solutions were prepared with a measurement solution (pH 5.7). The transient ion ﬂuxes were monitored for another 30 min in the meristem, elongation, and mature root zones, respectively. The same protocol was used to measure the NaCl-induced transient K^+^ flux kinetics in the roots of 2*x* and tetraploid (4*x*) *A. thaliana* (Columbia).

### Steady-state K^+^ and Na^+^ flux measurements

The root segments with 3 cm apices were sampled from control or NaCl-treated 2*x* and 6*x I. trifida* (24 h and 5 d after NaCl treatment). The roots were then transferred to the measuring chamber containing 10 ml of fresh measurement solution and were immobilized at the bottom. K^+^ and Na^+^ ﬂuxes were monitored in the following measurement solutions (Sun *et al*., 2009*a*, *b*; Yu *et al.*, 2016, 2018): K^+^: 150 mM NaCl, 0.1 mM MgCl_2_, 0.1 mM CaCl_2_, and 0.5 mM KCl, pH 5.7; Na^+^: 0.1 mM NaCl, 0.1 mM MgCl_2_, 0.1 mM CaCl_2_, and 0.5 mM KCl, pH 5.7. For Na^+^ ﬂux recording, the roots were rinsed with the measurement solution and immediately incubated in Na^+^ measurement solution to equilibrate for 30 min to decrease the effect of Na^+^ released from the surface of salt-stressed roots (Sun *et al.*, 2009*b*). The abrupt removal of 150 mM NaCl may potentially cause a hypo-osmotic shock and non-specific leak of Na^+^ (Cuin *et al.*, 2011). Our data proved that the Na^+^ efflux rates were similar when measured in the hypo-osmotic solution (measurement solution) and iso-osmotic solution (measurement solution containing 280 mM sorbitol) 30 min after the removal of 150 mM NaCl stress (Supplementary Fig. S3). For K^+^ ﬂux recording, 150 mM NaCl was added to the measurement solution to mimic a saline environment (Sun *et al*., 2009*a*, *b*; Yu *et al*., 2016, 2018). Ion ﬂuxes were determined along the root axis in three regions: meristem zone (300–600 µm from the tip with a measurement interval of 100 µm), elongation zone (1–3 mm from the tip with a measurement interval of 500 µm), and mature zone (10–15 mm from the tip with a measurement interval of 1 mm). Continuous recording was performed for 2–3 min at each measuring point in the three root zones. Steady-state ion ﬂuxes were expressed as the mean of several measuring points in each root zone.

### Pharmacology

Root segments collected from control plants were pre-treated with diphenylene iodonium (DPI, 100 μM) for 60 min prior to application of NaCl. Then, the transient K^+^ flux kinetics at the meristem root zone were recorded as described above. In addition, the Ca^2+^ flux was recorded at the elongation and mature root zones after 15 min of NaCl stress.

For prolonged salinity, 100 μM DPI, 10 mM EGTA, or 100 μM amiloride was added to the NaCl solution at the beginning of treatment. After 24 h and 5 d of treatment, the root segments were collected for steady-state Na^+^ or K^+^ flux measurements as described above. In addition, for DPI and EGTA treatment, the intracellular Na^+^ fluorescence in the mature root region of 6*x I. trifida* was also detected.

### Statistical analysis

Data were subjected to ANOVA. Significant differences between means were determined by using Duncan’s multiple range test. Unless otherwise stated, differences at *P*<0.05 were considered significant.

## Results

### Differential salt sensitivity and K^+^/Na^+^ homeostasis was observed in 2*x* and 6*x I. trifida* under salinity stress

**Fig. 1.**
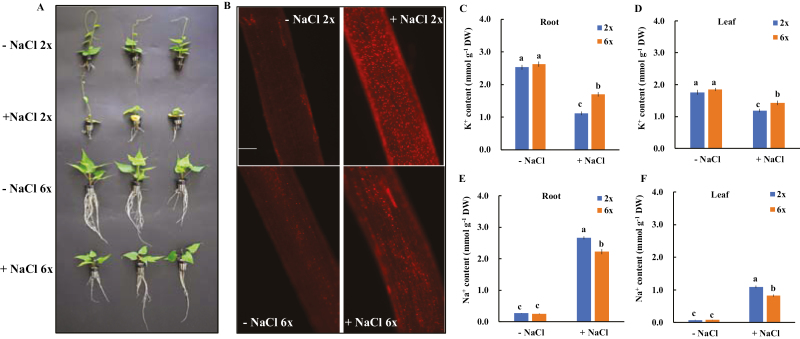
Effects of NaCl stress on the growth (A), root cell membrane integrity (B), tissue K^+^ (C and D), and Na^+^ (E and F) contents in 2*x* and 6*x I. trifida.* – NaCl indicates that plants were grown in the non-buffered culture solution; + NaCl indicates that plants were exposed to 150 mM NaCl stress for 7 d. (B) Representative PI staining image of the root elongation zone (three independent experiments) for each treatment; scale bar=0.2 mm. (C–F) Data were expressed as the mean ±SE (three independent experiments). Columns with different letters are significantly different at *P*<0.05.

In the absence of salinity stress, 6*x I. trifida* exhibited faster root growth rate and larger biomass productivity than 2*x I. trifida*, although the root length and morphology at the beginning of the experiment were similar (Fig. 1A; Supplementary Fig. S2). NaCl treatment (150 mM) for 7 d considerably delayed the growth of 2*x* and 6*x I. trifida*; however, leaf senescence and chlorosis were only observed in 2*x I. trifida* (Fig. 1A). In addition, the survival rate of 6*x I. trifida* (~90%) was markedly higher than that of 2*x I. trifida* (~10%) after 14 d of NaCl stress (data not shown). The PI staining experiment revealed the distinctive root cell membrane integrity of 2*x* and 6*x I. trifida* upon salt stress (Fig. 1B). After 7 d of 150 mM NaCl treatment, the root elongation zone (2–3 mm from the tip) of 2*x I. trifida* showed a strong red fluorescence in the nucleus of most cells, indicating that the PM integrity in this root region was damaged. However, the PI-stained nucleus in the same position of 6*x I. trifida* was substantially smaller than that of 2*x I. trifida* under saline conditions (Fig. 1B). These results showed that 2*x I. trifida* is more sensitive to salinity stress than 6*x I. trifida*.

NaCl treatment for 7 d substantially decreased the K^+^ content but increased the Na^+^ content in root and leaf tissues of 2*x* and 6*x I. trifida* (Fig. 1C–F). The K^+^ content in root and leaf tissues decreased by 56% and 32% in salinized 2*x I. trifida* and by 35% and 22% in salinized 6*x I. trifida*, respectively (Fig. 1C, D). The Na^+^ content in the root and leaf tissues of the salinized 2*x I. trifida* (2.4 mmol g^−1^ and 1.1 mmol g^−1^ DW, respectively) was remarkably higher than that of 6*x I. trifida* (2.0 mmol g^−1^ and 0.7 mmol g^−1^ DW, respectively) (Fig. 1E, F). These results suggest that 6*x I. trifida* possessed better capacity to regulate K^+^/Na^+^ homeostasis than 2*x I. trifida*.

### K^+^ and H^+^ flux kinetics in the root meristem, elongation, and mature zones upon exposure to salinity stress

**Fig. 2.**
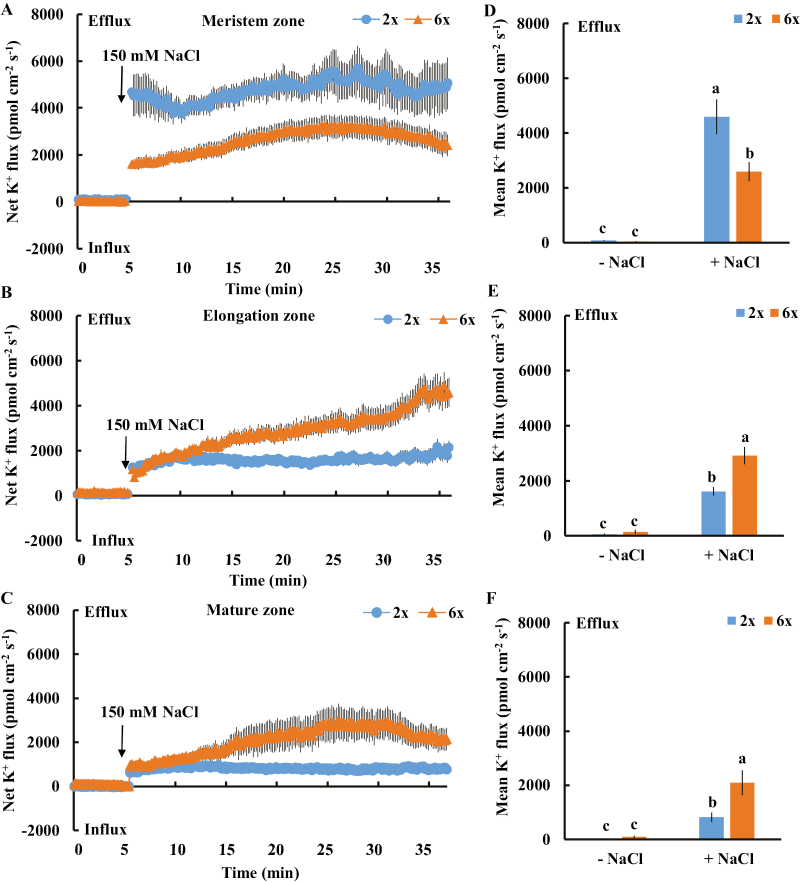
Effects of NaCl stress (150 mM) on the transient net K^+^ flux kinetics at the meristem (A: 500 µm from the tip), elongation (B: 3 mm from the tip), and mature (C: 15 mm from the tip) root zones in 2*x* and 6*x I. trifida*. Each point represents the mean of 20 roots collected from 10 individual plants. (D–F) Columns show the mean rate of K^+^ ﬂux before the addition of NaCl (~5 min) and after the addition of NaCl (~30 min). Different letters denote a significant differences at *P*<0.05.

**Fig. 3.**
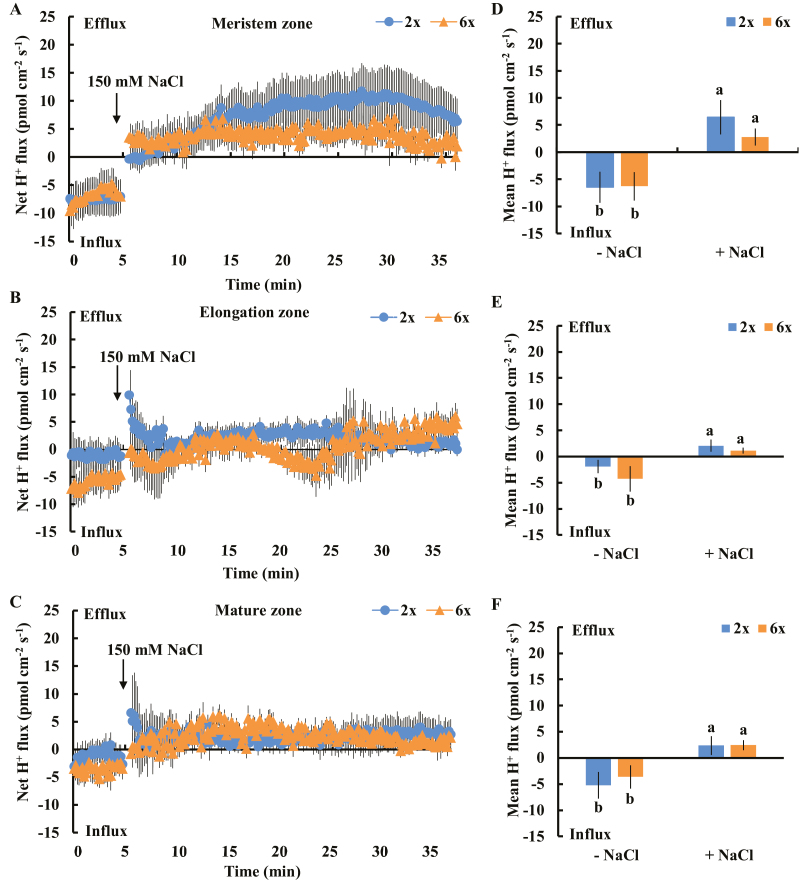
Effects of NaCl stress (150 mM) on the transient net H^+^ flux kinetics at the meristem (A: 500 µm from the tip), elongation (B: 3 mm from the tip), and mature (C: 15 mm from the tip) root zones in 2*x* and 6*x I. trifida*. Each point represents the mean of 20 roots collected from 10 individual plants. (D–F) Columns show the mean rate of H^+^ ﬂux before the addition of NaCl (~5 min) and after the addition of NaCl (~30 min). Different letters denote a significant difference at *P*<0.05.

An immense K^+^ efflux was observed in the 150 mM NaCl-treated meristem, elongation, and mature root zones of 2*x* and 6*x I. trifida*; however, the pattern differed considerably among the three root zones (Fig. 2A–C). In the meristem zone, a higher K^+^ efflux was observed in 2*x*, and the mean rate of salt-induced K^+^ leakage during salt exposure (~30 min) reached up to 4800 pmol cm^−2^ s^−1^ (1.9-fold higher than that of 6*x I. trifida*). However, an opposite trend was observed in the elongation and mature root zones, wherein 6*x I. trifida* exhibited a more acute salt-triggered K^+^ efflux than 2*x I. trifida*. The mean rates of K^+^ efflux in the elongation and mature root zones were 2900 pmol cm^−2^ s^−1^ and 2100 pmol cm^−2^ s^−1^ for 6*x I. trifida* and 1600 pmol cm^−2^ s^−1^ and 800 pmol cm^−2^ s^−1^ for 2*x I. trifida*, respectively (Fig. 2D–F). These two root zones exhibited a considerable difference between the tested ploidy (*P*<0.05). We tested the H^+^ flux kinetics in different root zones of 2*x* and 6*x I. trifida* in response to salinity stress. The onset of NaCl stress led to a remarkable shift in H^+^ influx toward obvious efflux in all root zones and ploidies tested (Fig. 3), which suggests the activation of PM H^+^-ATPase activity. However, the magnitude of this shift did not show a remarkable ploidy and root zone dependence (Fig. 3).

**Fig. 4.**
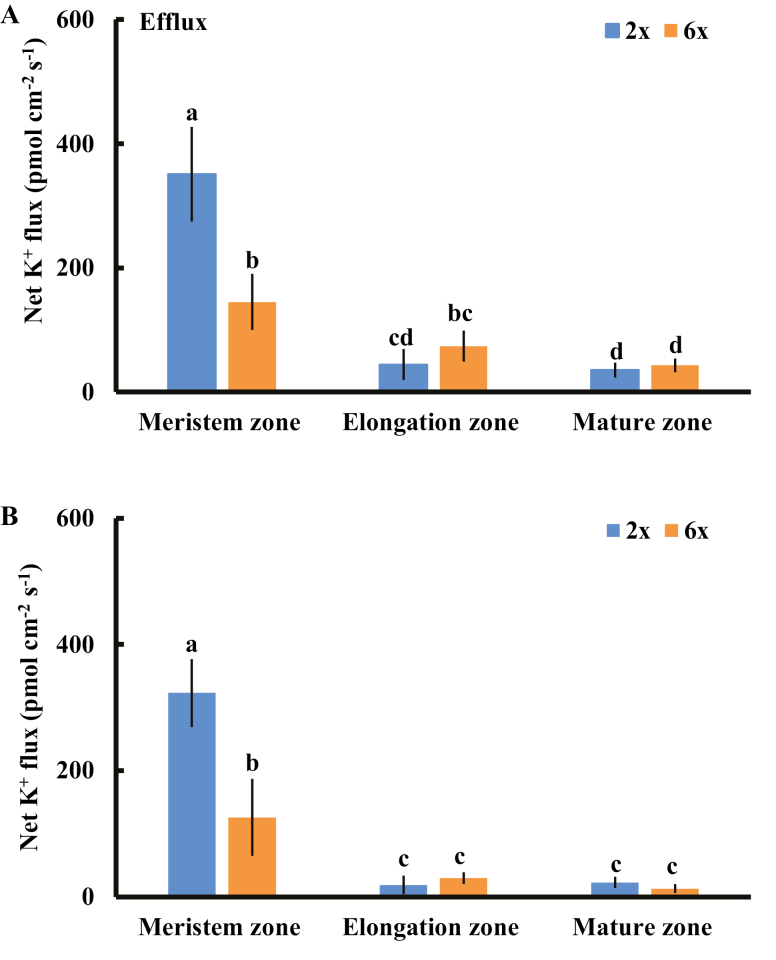
Effects of NaCl stress (150 mM) on the steady-state ﬂux of K^+^ at the different root regions of 2*x* and 6*x I. trifida*. The steady-state K^+^ ﬂux was measured from the meristem (300–600 µm from the tip), elongation (1–3 mm from the tip), and mature (10–15 mm from the tip) root zones after 24 h (A) and 5 d (B) of NaCl treatment. Each column is equivalent to the mean of 12 roots collected from six individual seedlings. The bars represent the SE of the mean. Columns labeled with different letters indicate a significant difference at *P*<0.05.

We measured the K^+^ ﬂux responses under prolonged salinity conditions to test whether the K^+^ efflux observed in transient flux recording experiments is temporary. After 24 h of 150 mM NaCl stress, we detected a steady K^+^ efflux in the meristem root zone in both 2*x* and 6*x I. trifida*; however, this efflux was considerably smaller than that in transient experiments (Fig. 4A). The rate of K^+^ efflux in this root zone of 2*x I. trifida* was remarkably higher than that of 6*x I. trifida* (Fig. 4A). The K^+^ efflux in the elongation and mature root zones of 2*x* and 6*x I. trifida* was much smaller, and no difference between the two plants was observed (Fig. 4A). The same trend was observed after 5 d of NaCl stress (Fig. 4B). All root zones of 2*x* and 6*x I. trifida* exhibited a minimal K^+^ influx in the absence of NaCl stress (Supplementary Fig. S4).

### Effect of diphenylene iodonium on the NaCl-induced K^+^ efflux from the meristem root zone of 2*x* and 6*x I. trifida*

**Fig. 5.**
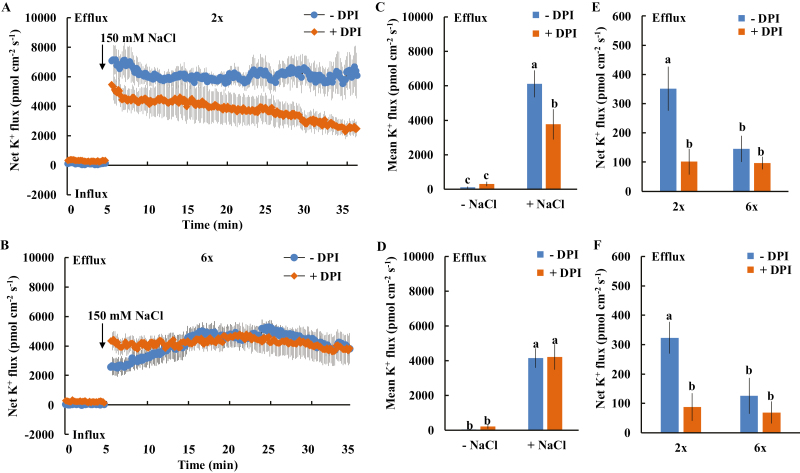
Effects of DPI on the NaCl-triggered K^+^ efflux in the meristematic root zone of 2*x* and 6*x I. trifida.* (A and B) Transient K^+^ flux kinetics measured at 500 µm from the tip. Each point represents the mean of the 10 roots collected from five individual plants. (C and D) Columns show the mean rate of K^+^ ﬂux before the addition of NaCl (~5 min) and after the addition of NaCl (~30 min). Different letters denote significant differences at *P*<0.05. (E and F) Steady-state K^+^ flux after prolonged NaCl stress (measured at 300–600 µm from the tip; E, 24 h; F, 5 d). Each column is equivalent to the mean of 10 roots collected from five individual seedlings. The bars represent the SE. Different letters denote significant differences at *P*<0.05.

Given that the differential K^+^ efflux upon salt shock in the elongation and mature root zones is temporary (Figs 2, 4), we explored the mechanism of salt-induced K^+^ efflux in the meristem root zone. In addition to PM H^+^-ATPase activity-regulated DA-KORCs the ROS-activated K^+^-permeable channels are also important in mediating salinity stress-triggered K^+^ efflux (Shabala *et al.*, 2016*b*). Pre-treatment of roots with DPI (a known inhibitor of PM NADPH oxidase; Sun *et al.*, 2010; Niu *et al.*, 2018) for 60 min considerably inhibited the NaCl stress-induced transient K^+^ efflux from the meristem root zone of 2*x I. trifida* (Fig. 5A). The mean rate of K^+^ efflux during salt exposure was reduced by 38% in the presence of DPI (Fig. 5C). Of note, the DPI inhibition of K^+^ efflux was more pronounced during the later period of salt shock (Fig. 5A; >60% inhibition during the last 10 min of recording). However, DPI treatment did not alter the salt shock-induced K^+^ efflux from the meristem root region of 6*x I. trifida* (Fig. 5B, D). In addition, it also inhibited the prolonged salinity- (24 h and 5 d) induced K^+^ efflux from the meristem root zone in 2*x I. trifida* but not in 6*x I. trifida* (Fig. 5E, F).

### H_2_O_2_ production in different root zones of 2*x* and 6*x I. trifida* under salinity stress

**Fig. 6.**
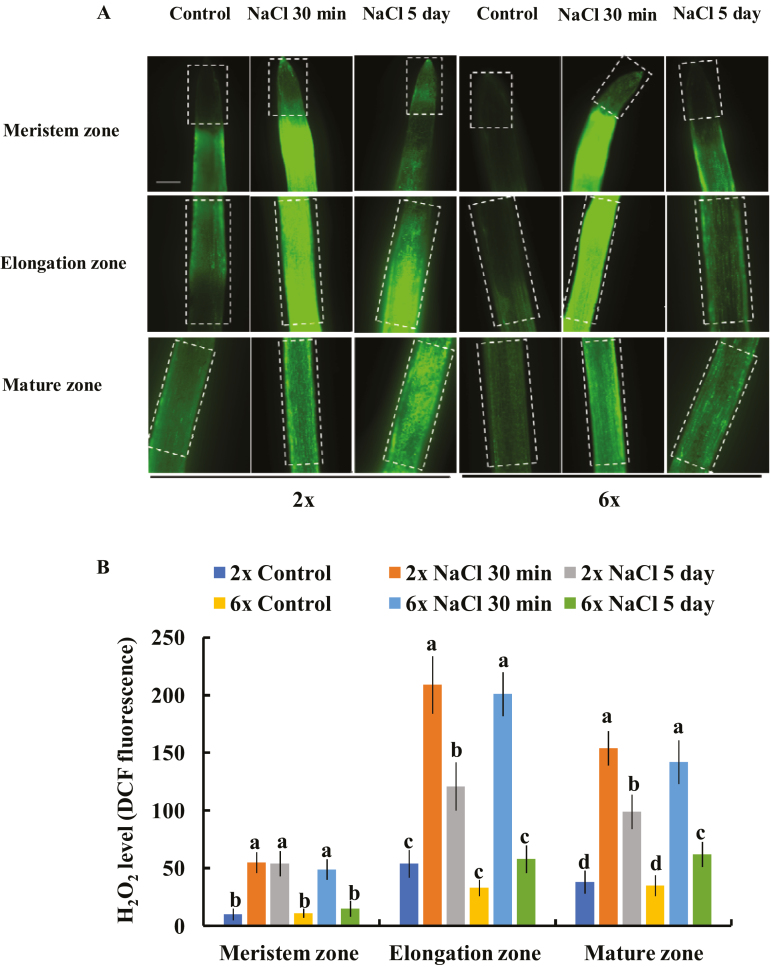
Effects of NaCl stress (150 mM NaCl) on the H_2_O_2_ production in the meristem, elongation, and mature root zones of 2*x* and 6*x I. trifida*. (A) Representative images showing the alteration of H_2_O_2_ accumulation before and after NaCl treatment. Boxes indicate the AOI for the quantification of fluorescence intensity by using Image-Pro Plus 6.0. (B) DCF fluorescence intensity in (A). For each treatment, 20 root segments from 10 individual plants were observed and quantified. Columns labeled with different letters indicate significant difference at *P*<0.05.

We measured the salt-triggered H_2_O_2_ accumulation in different root zones of 2*x* and 6*x I. trifida*. Root treatment with 150 mM NaCl for 30 min resulted in a substantial accumulation of H_2_O_2_ in the meristem, elongation, and mature root zones of 2*x* and 6*x I. trifida*, as indicated by the obvious DCF fluorescence (Fig. 6A). This salt-induced rapid accumulation of H_2_O_2_ was highly tissue specific. The highest accumulation of H_2_O_2_ was found in the elongation zone, whereas the lowest accumulation was observed in the meristem zone (Fig. 6A, B). However, the rapid increase of H_2_O_2_ triggered by salinity stress was not related to the ploidy level in *I. trifida* (Fig. 6A, B). The accumulation of H_2_O_2_ in the three root zones of 2*x I. trifida* was considerably higher than that of 6*x I. trifida* under prolonged salinity stress (5 d; Fig. 6A, B).

### Differential sensitivity of K^+^-permeable channels to H_2_O_2_ and OH· was observed in a root-zone-specific manner in 2*x* and 6*x I. trifida*

**Fig. 7.**
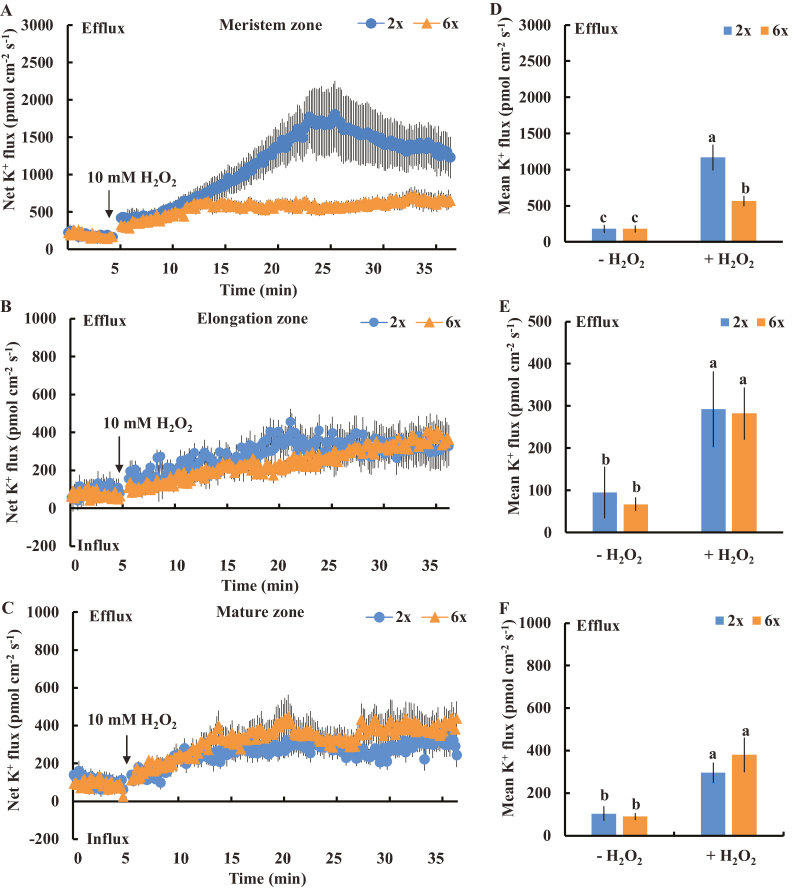
Effects of H_2_O_2_ (10 mM) on the transient net K^+^ flux kinetics at the meristem (A: 500 µm from the tip), elongation (B: 3 mm from the tip), and mature (C: 15 mm from the tip) root zones in 2*x* and 6*x I. trifida*. Each point represents the mean of 12 roots collected from six individual plants. (D–F) Columns show the mean rate of K^+^ ﬂux before the addition of H_2_O_2_ (~5 min) and after the addition of H_2_O_2_ (~30 min). Different letters denote significant differences at *P*<0.05.

We measured the H_2_O_2_-induced K^+^ flux kinetics in the meristem, elongation, and mature root zones to test the sensitivity of root K^+^-permeable channels to ROS. In the meristem zone, triphasic K^+^ flux kinetics were recorded in 2*x* (Fig. 7A). The initial H_2_O_2_-induced K^+^ efflux (the first 10 min of H_2_O_2_ treatment) in 2*x I. trifida* did not show any significant difference compared with that of 6*x I. trifida*. However, a drastic increase in K^+^ efflux was recorded in 2*x I. trifida* during the second 10 min of H_2_O_2_ treatment. This efflux gradually decreased in the last 10 min of recording. However, this pattern was not recorded in 6*x I. trifida* (Fig. 7A). The mean rate of H_2_O_2_-triggered K^+^ efflux reached 1200 pmol cm^−2^ s^−1^ in the meristem root zone of 2*x I. trifida* (2.1-fold higher than that of 6*x I. trifida*). The H_2_O_2_-triggered K^+^ efflux was also recorded in the elongation and mature root zones; however, the magnitude of K^+^ efflux was considerably lower than that in the meristem zone (Fig. 7B, C). In the two root zones, the H_2_O_2_-triggered K^+^ flux kinetics and mean rate of K^+^ efflux were similar between 2*x* and 6*x I. trifida* (Fig. 7B, C, E, F).

**Fig. 8.**
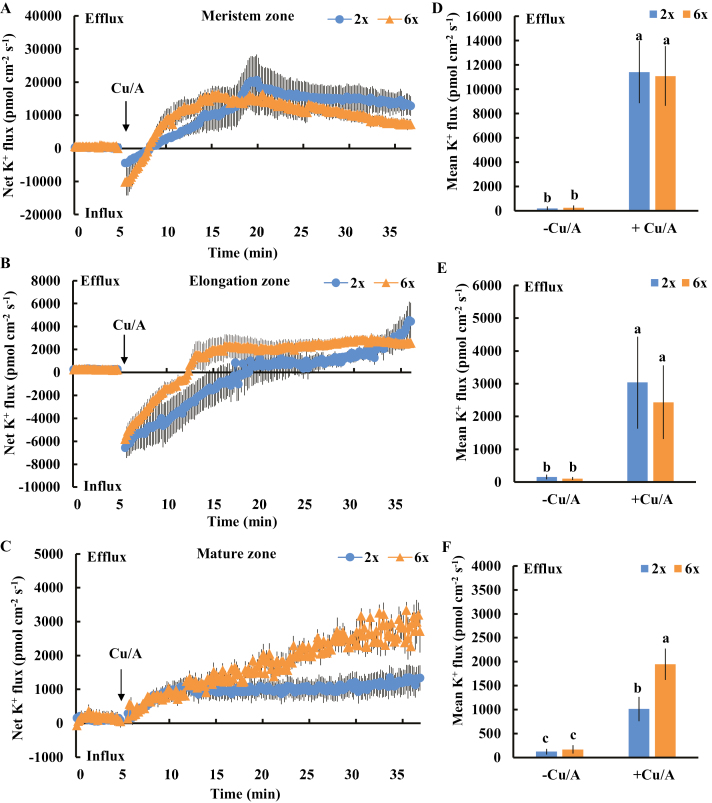
Effects of OH· (1 mM Cu/A) on the transient net K^+^ flux kinetics at the meristem (A: 500 µm from the tip), elongation (B: 3 mm from the tip) and mature (C: 15 mm from the tip) root zones in 2*x* and 6*x I. trifida*. Each point represents the mean of 12 roots collected from six individual plants. (D–F) Columns show the mean rate of K^+^ ﬂux before the addition of Cu/A (~5 min) and after the addition of Cu/A (~30 min). Different letters denote significant differences at *P*<0.05.

The addition of H_2_O_2_ to the roots may result in the formation of OH·, which can activate a range of cation-permeable ion channels in plants (Demidchik *et al.*, 2018). Thus, we tested the sensitivity of root K^+^-permeable channels to OH·. The addition of an OH·-generating mixture (1 mM Cu/A) to the roots led to a rapid influx of K^+^ in the meristem and elongation root zones of both 2*x* and 6*x I. trifida*. Then, this influx gradually shifted toward a drastic efflux (Fig. 8A, B). In the meristem zone, the OH·-induced K^+^ flux kinetics and mean rate of K^+^ efflux during the stimulation were similar between the 2*x* and 6*x I. trifida* (Fig. 8A, D). Of note, the transition time of K^+^ flux in the elongation root zone was shorter in 6*x I. trifida* than in 2*x I. trifida*. The magnitude of K^+^ efflux in 6*x I. trifida* was also higher than that in 2*x I. trifida*, although the mean rate of K^+^ efflux did not exhibit any significant difference between the two plants (Fig. 8B, E). Interestingly, an instantaneous K^+^ efflux was observed in the mature root zone after Cu/A application, and the magnitude of this efflux in 6*x I. trifida* was remarkably higher than that in 2*x I. trifida* (Fig. 8C, F). These results suggest that the K^+^-permeable channels in the elongation and mature root zones of 6*x I. trifida* were more sensitive to OH· than those of 2*x I. trifida*. However, the K^+^-permeable channels in the meristem root zone of 2*x I. trifida* were more sensitive to H_2_O_2_ than those of 6*x I. trifida*.

### Differential Ca^2+^ flux patterns upon H_2_O_2_ and NaCl exposure were observed in elongation and mature root zones in 2*x* and 6*x I. trifida*

**Fig. 9.**
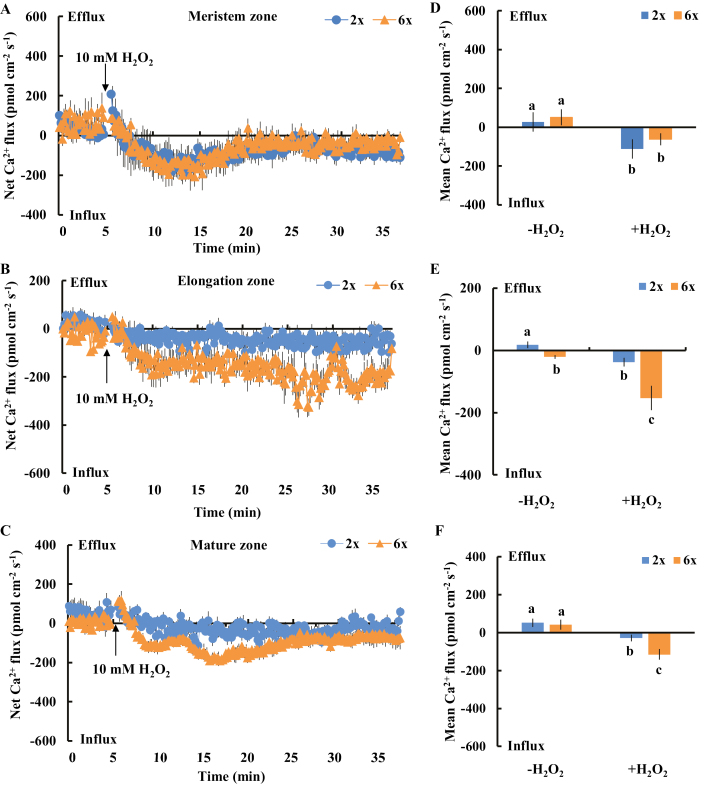
Effects of H_2_O_2_ (10 mM) on the transient net Ca^2+^ flux kinetics at the meristem (A: 500 µm from the tip), elongation (B: 3 mm from the tip), and mature (C: 15 mm from the tip) root zones in 2*x* and 6*x I. trifida*. Each point represents the mean of 12 roots collected from six individual plants. (D–F) Columns show the mean rate of Ca^2+^ ﬂux before the addition of H_2_O_2_ (~5 min) and after the addition of H_2_O_2_ (~30 min). Different letters denote significant differences at *P*<0.05.

The ROS-triggered K^+^ efflux across the PM in plant roots is generally accompanied by a Ca^2+^ influx, which is essential for intracellular signaling and salt adaption (Wang *et al.*, 2018). Thus, we tested the sensitivity of PM Ca^2+^-permeable channels to H_2_O_2_ in different root zones. The addition of 10 mM H_2_O_2_ to the roots triggered an immediate influx of Ca^2+^ regardless of cell type and ploidy (Fig. 9). The Ca^2+^ flux pattern and mean rate of Ca^2+^ influx during H_2_O_2_ stimulation in the meristem root zone were similar in the 2*x* and 6*x I. trifida* (Fig. 9A, D). Intriguingly, the H_2_O_2_-triggered Ca^2+^ influx in the elongation and mature root zones of 6*x I. trifida* was substantially higher than that of 2*x I. trifida* (Fig. 9B, C, E, F), suggesting the high sensitivity of Ca^2+^-permeable channels to H_2_O_2_ in the two root zones of 6*x I. trifida*. We did not detect a remarkable difference in OH·-triggered Ca^2+^ flux patterns between 2*x* and 6*x I. trifida* (Supplementary Fig. S5).

**Fig. 10.**
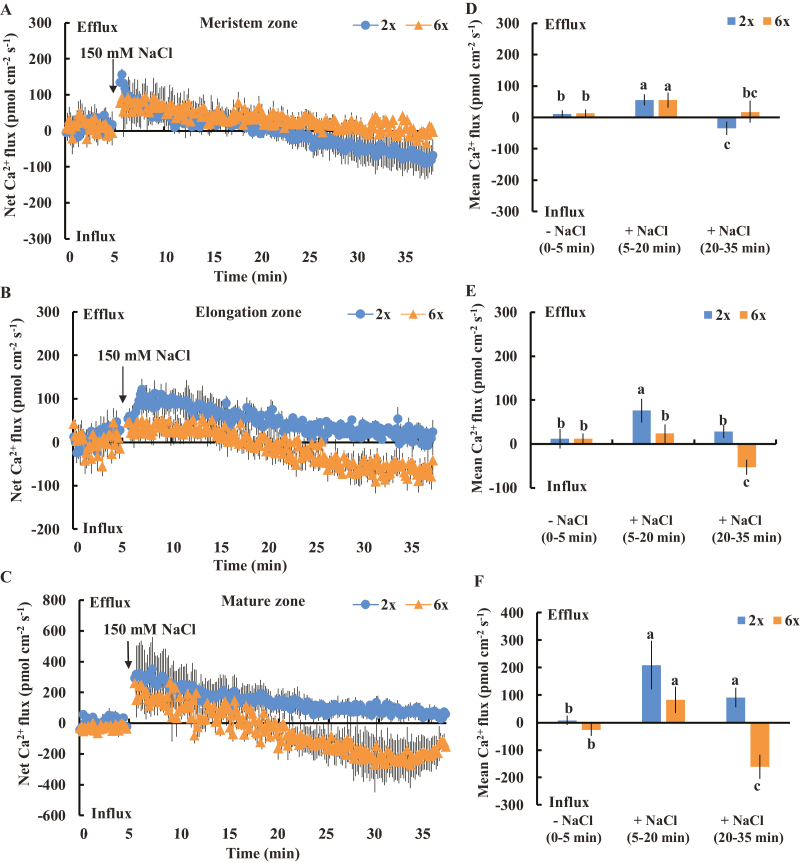
Effects of NaCl stress (150 mM) on the transient net Ca^2+^ flux kinetics at the meristem (A: 500 µm from the tip), elongation (B: 3 mm from the tip), and mature (C: 15 mm from the tip) root zones in 2*x* and 6*x I. trifida*. Each point represents the mean of 12 roots collected from six individual plants. (D–F) Columns show the mean rate of Ca^2+^ ﬂux before the addition of NaCl (~5 min), the early period of NaCl stress (~15 min), and the later period of NaCl stress (~15 min). Different letters denote significant differences at *P*<0.05.

Given that an early salt-induced H_2_O_2_ increase and differential sensitivity of PM Ca^2+^ channels to H_2_O_2_ in the elongation and mature root zones were observed in 2*x* and 6*x I. trifida*, the differential Na^+^ homeostasis in these regions may be causally related to H_2_O_2_-dependent Ca^2+^ signaling. Consistent with this hypothesis, a differential Ca^2+^ flux pattern upon NaCl treatment was recorded in 2*x* and 6*x I. trifida*. In the meristem zone, the NaCl-triggered Ca^2+^ flux kinetics were biphasic, which comprised an instantaneous and gradually decreased Ca^2+^ efflux (0–15 min after NaCl addition) and an obvious transition of Ca^2+^ efflux toward Ca^2+^ influx (15–30 min after NaCl addition) (Fig. 10A). However, we did not observe a significant difference in the Ca^2+^ flux pattern between 2*x* and 6*x I. trifida* (Fig. 10D). In the elongation and mature zones, an obvious transition of NaCl-induced Ca^2+^ efflux toward Ca^2+^ influx was recorded in 6*x* but not in 2*x I. trifida* (Fig. 10B, C, E, F). The mean rate of Ca^2+^ influx during the late period of recording (15–30 min after NaCl addition) was remarkably higher in 6*x* than in 2*x I. trifida* (Fig. 10E, F). This NaCl-induced Ca^2+^ influx in the two root zones of 6*x I. trifida* was considerably inhibited in the presence of DPI, indicating the activation of PM Ca^2+^-permeable channels by H_2_O_2_ (Supplementary Fig. S6).

### Na^+^ efflux and intracellular Na^+^ accumulation in the roots of 2*x* and 6*x I. trifida*

**Fig. 11.**
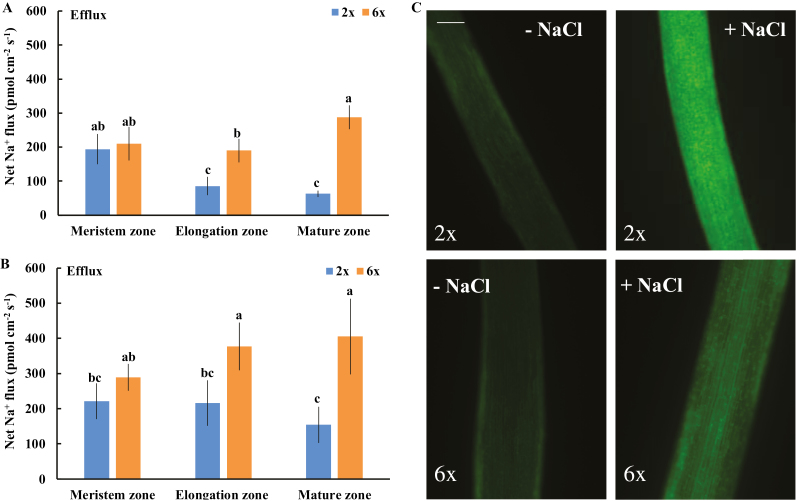
Effects of NaCl stress (150 mM) on the steady-state ﬂux of Na^+^ and intracellular Na^+^ accumulation in roots of 2*x* and 6*x I. trifida*. (A and B) Na^+^ flux. The steady-state Na^+^ ﬂux was measured from the meristem (300–600 µm from the tip), elongation (1–3 mm from the tip), and mature (10–15 mm from the tip) root zones after 24 h (A) and 5 d (B) of NaCl treatment. Each column is equivalent to the mean of 12 roots collected from six individual seedlings. The bars represent the SE. Columns labeled with different letters indicate a significant difference at *P*<0.05. (B) Na^+^ accumulation in the mature root zone as visualized by the CoroNa-Green ﬂuorescent dye after 5 d of 150 mM NaCl treatment. A typical image (of 20) is shown. All images were taken using the same settings and exposure times to enable direct comparisons. Sale bar in (C)=0.3 mm.

After 24 h and 5 d of NaCl stress (150 mM), an obvious Na^+^ efflux was recorded in all tested root zones of 2*x* and 6*x I. trifida* (Fig. 11A, B), reflecting the activation of Na^+^ extrusion activity by NaCl. However, the Na^+^ efflux in the elongation and mature root zones of 6*x I. trifida* was higher (2.5- to 4.5-fold) than that of 2*x I. trifida* (Fig. 11A, B). The Na^+^ efflux in the two root zones of 6*x I. trifida* was inhibited in the presence of amiloride, which is an inhibitor of Na^+^/H^+^ antiporter (Supplementary Fig. S7; Sun *et al.*, 2009*b*). Consistent with the Na^+^ extrusion activity, a higher accumulation of intracellular Na^+^ was observed in mature root cells of 2*x I. trifida* than in those of 6*x I. trifida* (Fig. 11C). The Na^+^ extrusion activity and intracellular accumulation of Na^+^ in mature root cells of 6*x I. trifida* were markedly decreased and enhanced in the presence of DPI and EGTA (Supplementary Fig. S8), which indicates the involvement of salt-induced H_2_O_2_ production and extracellular Ca^2+^ influx in mediating Na^+^ homeostasis.

## Discussion

### An enhanced capacity for mediation of K^+^/Na^+^ homeostasis contributed to the salt tolerance of 6*x I. trifida*

Genetically, the variation in plant salt tolerance is associated with the ploidy level of the species (Ashraf and McNeilly, 2004). Polyploids possess improved ability to accumulate K^+^ and exclude Na^+^ under saline conditions, which could contribute to the superior salt adaptation of polyploid populations (Chao *et al.*, 2013; Yang *et al.*, 2014). Maintaining an appropriate K^+^/Na^+^ homeostasis at the cellular and tissue levels is an important salt-resistant trait in plants (Munns and Tester, 2008; Anschütz *et al.*, 2014; Deinlein *et al.*, 2014). However, the mechanism for this ploidy-determined K^+^/Na^+^ homeostasis remains unclear. In the present study, we revealed the differential salt sensitivities of 2*x* and 6*x I. trifida*. The growth phenotype and PM integrity data suggest that the salt adaptation ability of 6*x I. trifida* was better than that of its 2*x* ancestor (Fig. 1).

Given the substantial uptake of Na^+^ upon salt stress and the strong cytotoxicity of Na^+^, plant survival under saline conditions is critically dependent on the ability to restrict Na^+^ accumulation in the cytosol (Tester and Davenport, 2003; Munns and Gilliham, 2015). The maintenance of high cytosolic K^+^ levels under saline conditions is essential for enzymatic activities, appropriate metabolic processes, ionic homeostasis, charge balance, and the prevention of cell death induced by high salinity (Shabala and Cuin, 2008; Shabala, 2009; Demidchik *et al.*, 2010). A causality between the superior ability for K^+^ retention (especially in roots) and plant salt tolerance has been established in various species (Chen *et al.*, 2005; Cuin *et al.*, 2008; Sun *et al.*, 2009*a*; Bose *et al.*, 2015; Chakraborty *et al.*, 2016; Yu *et al.*, 2016; Zeng *et al.*, 2018). Here, we showed that under saline conditions, 6*x I. trifida* possessed better capacity for the maintenance of K^+^ levels and restriction of Na^+^ accumulation in root and leaf tissues than 2*x I. trifida* (Fig. 1C–F). These findings agree with a previous observation that polyploids maintain a more appropriate K^+^/Na^+^ homeostasis under saline conditions (Chao *et al.*, 2013; Yang *et al.*, 2014). Thus, the strong ability to regulate K^+^/Na^+^ homeostasis can explain the ploidy-dependent variation in the salinity tolerance of *I. trifida.* The physiological mechanisms for the superior K^+^/Na^+^ homeostasis in salinized 6*x I. trifida* include the following: (i) the low sensitivity of PM K^+^-permeable channels to H_2_O_2_ in the meristem root zone improved K^+^ retention; and (ii) the high sensitivity of PM Ca^2+^-permeable channels to H_2_O_2_ in the elongation and mature root zones contributed to the strong activity of Na^+^/H^+^ antiporter across the PM.

### Lower sensitivity of PM K^+^-permeable channels to H_2_O_2_ in the meristem root zone provided the superior K^+^ retention ability in salinized 6*x I. trifida*

A comprehensive analysis of cell type-specific sensitivity to salinity stress in root tissues revealed that the salt-induced K^+^ efflux in the root apex, including meristem and elongation root zones, determined the overall root K^+^ status and salt tolerance in salinized barley (Shabala *et al.*, 2016*b*). Our results strongly suggest that the magnitude of salt-induced K^+^ efflux from the meristem root zone controls the ploidy-determined K^+^ retention in *I. trifida* (Figs 2A, 4). The root-zone-specific K^+^ flux patterns under saline conditions strongly support this viewpoint (Figs 2, 4). Vacuolar K^+^ release compensates for the cytosolic K^+^ loss caused by salinity stress in vacuolated cells (Shabala and Pottosin, 2014). Thus, the high and continuous efflux of K^+^ from root meristematic epidermis cells may be highly detrimental for meristem size control, stem cell activity, cell division, cell cycle progression, root growth, and metabolic processes in 2*x I. trifida* (West *et al.*, 2004; Liu *et al.*, 2015). One may question how the small volume of the meristem root zone contributes to the differential K^+^ loss at the whole-root level. To regain the optimal K^+^ level and physiological activity of actively dividing cells, a compensatory transport of K^+^ from proximal cells in the longitudinal direction may be triggered by drastic K^+^ loss in the meristem cells, thereby causing K^+^ deprivation at the whole-root level (Shabala, 2017). The salt-induced differential K^+^ flux kinetics were not recorded from root tissues in 2*x* and 4*x* Arabidopsis (Supplementary Fig. S9). An enhanced K^+^ acquisition in the roots may operate in 4*x* Arabidopsis to achieve superior K^+^ nutrition under salinity stress (Chao *et al.*, 2013). This inconsistency indicates that the ploidy-dependent regulatory mechanisms of K^+^ retention under saline conditions are diverse across different plant species.

PM H^+^-ATPase activity, which is usually reflected by the kinetics and magnitude of H^+^ efflux in plant cells (Bose *et al.*, 2013; Yu *et al.*, 2016; Han *et al.*, 2017), is essential for the maintenance of membrane potential across the PM, prevents K^+^ loss through DA-KORCs, and contributes to the retention of high cytosolic K^+^ levels under saline conditions (Sun *et al.*, 2009*a*; Anschütz *et al.*, 2014; Yu *et al*., 2016, 2018). The intrinsically high H^+^-ATPase activity in specific root zones contributes to the low salt-induced K^+^ efflux in barley (Shabala *et al.*, 2016*b*). However, the salt-induced H^+^ flux kinetics and H^+^ efflux rate did not differ from the corresponding root zones between 2*x* and 6*x I. trifida* (Fig. 3), indicating a similar activation of PM H^+^-ATPase activity upon exposure to salinity stress. Thus, the varying K^+^ efflux upon NaCl stress in 2*x* and 6*x I. trifida* roots was not ascribed to the difference in PM H^+^-ATPase activity.

Salinity stress generally induces a rapid increase in H_2_O_2_ in plant root cells, where PMs harbor various H_2_O_2_-activated K^+^-permeable channels (Sun *et al*., 2010*a*, 2012; Shabala *et al.*, 2015; Wang *et al.*, 2018). The H_2_O_2_ produced in the roots could interact with transition metals (Fe^2+^ or Cu^+^) to produce highly reactive OH·, which activates various K^+^-permeable channels, thereby causing massive K^+^ efﬂux (Demidchik *et al.*, 2010; Shabala and Pottosin, 2014). Approximately 60% of NaCl-induced K^+^ efﬂux in barley roots is ascribed to the NSCCs (Shabala *et al.*, 2016*b*), which can be activated by ROS (Demidchik *et al.*, 2010). The low sensitivity of K^+^-permeable channels to ROS confers the high salt tolerance in *Brassica* species (Chakraborty *et al.*, 2016). In the present study, a considerable DPI inhibition of NaCl-induced K^+^ efflux from meristematic cells was observed in 2*x* but not in 6*x I. trifida* (Fig. 4), suggesting that the excess K^+^ efflux in 2*x I. trifida* was ascribed to the ROS-activated K^+^-permeable channels. Although early ROS production induced by salt stress is considered an essential signal for triggering a cascade of adaptive responses (Sun *et al*., 2010, 2012; Mittler *et al.*, 2011; Ma *et al.*, 2012; Shabala *et al.*, 2015), it can be detrimental to plant cells upon exceeding a certain threshold, which is dependent on the species or tissue sensitivity (Sadhukhan *et al.*, 2017). The PM K^+^-permeable channels in the meristematic root zone of 2*x I. trifida* are more sensitive to H_2_O_2_ than those of 6*x I. trifida* as indicated by the ROS-induced K^+^ flux kinetics (Figs 7A, 8A). Given that the early salt-induced H_2_O_2_ production in the meristem zone is ploidy independent (Fig. 6), the excess K^+^ efflux in the meristematic root zone of 2*x I. trifida* possibly originated from the PM K^+^-permeable channels (maybe harbored in a large population) that are highly sensitive to salt-induced H_2_O_2_ production. This result agrees with a previous report that the magnitude of H_2_O_2_-induced K^+^ efflux in the mature root zone is associated with the salinity tolerance of barley (Wang *et al.*, 2018), although the H_2_O_2_-induced differential K^+^ efflux was only observed in the meristematic root zone in this study (Fig. 7). The H_2_O_2_ level in the root meristem zone of 2*x I. trifida* under prolonged salinity was considerably higher than that of 6*x I. trifida* (Fig. 6). This finding can be explained by the high endogenous level of antioxidant activity in the meristematic root zone of 6*x I. trifida*. The weak antioxidant activity in 2*x I. trifida* may accelerate the H_2_O_2_-stimulated K^+^ loss under long-term salinity (Shabala *et al.*, 2016*b*).

### Higher sensitivity of PM Ca^2+^-permeable channels to H_2_O_2_ in the elongation and mature root zones contributed to the robust Ca^2+^ influx and Na^+^ homeostasis in salinized 6*x I. trifida*

The exposure to salt stress increases the Na^+^/H^+^ antiporter activity across the PM in the root epidermis of different species (Sun *et al.*, 2009*b*; Cuin *et al.*, 2011; Niu *et al.*, 2018). The activity of PM Na^+^/H^+^ exchange is positively associated with the salt tolerance of plants (Munns and Tester, 2008; Deinlein *et al.*, 2014). Results show that 6*x I. trifida* was more efficient in extruding Na^+^ from the elongation and mature root zones than 2*x I. trifida* (Fig. 11). The amiloride inhibition of Na^+^ efflux in the two root zones of 6*x I. trifida* suggests that the detected Na^+^ exclusion probably resulted from the active Na^+^/H^+^ exchange across the PM (Sun *et al.*, 2009*b*; Cuin *et al.*, 2011; Niu *et al.*, 2018). Thus, the intracellular accumulation of Na^+^ in the mature root zone of 6*x I. trifida* was much lower than that of 2*x I. trifida* (Fig. 11). Given the importance of the mature root zone in mediating the Na^+^ radial transport to the xylem vessel (Plett and Møller, 2010), this ploidy-dependent and highly root-zone-specific Na^+^/H^+^ antiporter activity possibly contributed to the low Na^+^ accumulation in 6*x I. trifida* at the whole-plant level (Fig. 1E, F).

A well-accepted SOS signaling cascade is required for the activation of PM Na^+^/H^+^ antiporter activity in plants (Luan, 2009; Zhu, 2016). Here, a considerably higher Ca^2+^ influx was observed in the elongation and mature root zones in salinized 6*x I. trifida* than in 2*x I. trifida* (Fig. 10). Interestingly, a higher K^+^ efflux triggered by the same treatment was recorded at the corresponding root zones in 6*x I. trifida* (Fig. 2). However, this temporary K^+^ efflux may not be related to the K^+^/Na^+^ homeostasis regulation in 6*x I. trifida*. The higher and transient K^+^ efflux in 6*x I. trifida* may be correlated to the higher sensitivity of PM K^+^-permeable channels to OH· (Fig. 8) and play an important role in balancing the charge during Ca^2+^ influx upon exposure to salinity stress (Shabala *et al*, 2016*b*; Wang *et al.*, 2018). Thus, the robust Ca^2+^ influx upon exposure to NaCl stress may facilitate the [Ca^2+^]_cyt_ increment and the subsequent activation of the PM Na^+^/H^+^ antiport system through the SOS signaling pathway (Sun *et al*., 2010, 2012). This high Ca^2+^ influx may reflect the high Na^+^ exclusion activity in the two root zones of 6*x I. trifida* (Fig. 11). In addition, the temporary and high K^+^ efflux in the two root zones, especially in the elongation zone, in 6*x I. trifida* may act as a signal that will alter the metabolic processes and save energy for salt adaptation and repair (Shabala, 2017).

Salt-induced H_2_O_2_ production is a key signaling molecule involved in the mediation of Na^+^/H^+^ exchange across the PM through various mechanisms (Zhang *et al.*, 2007; Chung *et al.*, 2008; Sun *et al.*, 2010; Ma *et al.*, 2012; Niu *et al.*, 2018). We did not find a ploidy-dependent difference in the early H_2_O_2_ increase in elongation and mature root zones under salt stress (Fig. 6), suggesting that the amount of salt-induced H_2_O_2_ was not the main reason for the NaCl-induced differential Ca^2+^ flux kinetics. Interestingly, the PM Ca^2+^-permeable channels in the elongation and mature root zones of 6*x I. trifida* were highly sensitive to H_2_O_2_ based on our NMT data (Fig. 9). The Ca^2+^ influx mediated by the inward-rectifying NSCCs in the root elongation zone can be activated by H_2_O_2_ from either side of the membrane (Demidchik *et al.*, 2007, 2018). This high H_2_O_2_-induced Ca^2+^ influx in 6*x I. trifida* may be ascribed to the direct activation of PM Ca^2+^-permeable NSCCs (maybe harbored in a large population) to the external H_2_O_2_. In addition, the high sensitivity and permeability of PM aquaporin to H_2_O_2_ in 6*x I. trifida* may allow the rapid entry of H_2_O_2_ into the cells, thereby activating the PM Ca^2+^-permeable NSCCs inside the PM (Sadhukhan *et al.*, 2017). The Na^+^ efflux and Ca^2+^ influx decreased, whereas the intracellular Na^+^ accumulation increased in the two root zones of 6*x I. trifida* pre-treated with DPI under saline conditions (Supplementary Figs S6, S8). These results were consistent with a previous observation that DPI inhibited the Ca^2+^ transport and Na^+^ homeostasis in other species (Sun *et al.*, 2010; Niu *et al.*, 2018). Hence, the high sensitivity of PM Ca^2+^-permeable channels to H_2_O_2_ in the elongation and mature root zones contributes to the robust Ca^2+^ influx and excellent Na^+^ homeostasis in salinized 6*x I. trifida*.

### Conclusion

**Fig. 12.**
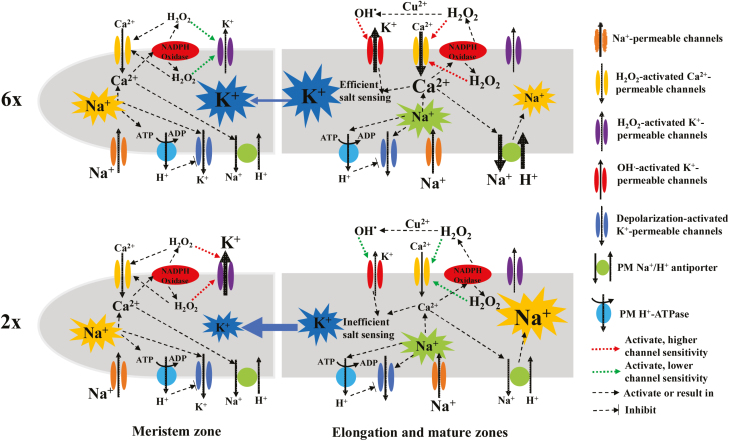
Schematic model of the mediation of root K^+^/Na^+^ homeostasis in the response of 2*x* and 6*x I. trifida* to NaCl stress (solid arrows indicate the ion flux direction and width reflects flux magnitude). In all root regions, NaCl stress results in a similar activation of PM H^+^-ATPase activity in 2*x* and 6*x I. trifida*, causing a similar K^+^ loss through depolarization-activated K^+^ channels. The increment in cytosolic Na^+^ triggers an elevation of cytosolic Ca^2+^ and stimulates NADPH oxidase activity, resulting in H_2_O_2_ accumulation within the cells or in the apoplast and activating a series of H_2_O_2_-specific K^+^- and Ca^2+^-permeable channels. In the meristem zone, the lower sensitivity of K^+^-permeable channels to H_2_O_2_ contributes to the lower K^+^ loss in 6*x I. trifida*. The drastic K^+^ loss in the meristem zone of 2*x I. trifida* may trigger the longitudinal transport of K^+^ to regain the optimal K^+^ level of the meristematic cells, thereby causing the K^+^ deprivation at the whole-root level. In the elongation and mature zones, the increased sensitivity of Ca^2+^-permeable channels to H_2_O_2_ causes a robust Ca^2+^ influx through the H_2_O_2_-activated Ca^2+^-permeable channels, thereby triggering stronger Na^+^/H^+^ antiport activity across the PM in 6*x I. trifida*. Charge imbalance caused by the Ca^2+^ influx may be offset by the OH^.^ -activated K^+^ efflux. Hence, the K^+^/Na^+^ homeostasis in root tissues is retained in salinized 6*x I. trifida*.

To the best of our knowledge, this study was the first attempt to investigate the relationship between the ploidy level of plants and root-zone-specific ion transport under saline conditions. The highly root-zone-specific sensitivity of PM K^+^- and Ca^2+^-permeable channels to H_2_O_2_ determines the superior capacity of K^+^/Na^+^ homeostasis in 6*x I. trifida* (Fig. 12), a potential ancestor of cultivated sweet potato (Feng *et al.*, 2018). Our results will provide information to understand how autohexaploids maintain better K^+^/Na^+^ homeostasis under saline conditions in plants. These novel physiological traits of 6*x I. trifida* may have potential application in the improvement of salt tolerance in cultivated sweet potato in the future. Additional studies are required to explore the molecular mechanisms underlying this highly cell-type-specific ion transport in the roots of 6*x I. trifida*. Considering that only one each of 2*x* and 6*x I. trifida* species were investigated, additional works are required to collect more genotypes of *I. trifida* with different ploidy levels. The comparison would be strengthened by assessing more genotypes from each ploidy.

## Supplementary data

Supplementary data are available at *JXB* online.

Fig. S1. Representative cytogenetic images show the mitotic metaphase of diploid (2*x*, 30 chromosomes) and hexaploid (6*x*, 90 chromosomes) *I. trifida*.

Fig. S2. Morphology of rooted seedlings of 2*x* and 6*x I. trifida*. Uniform seedlings were selected for experiments.

Fig. S3. Steady-state ﬂux of Na^+^ in the roots of 2*x* and 6*x I. trifida* measured in hypo-osmotic (measurement solution) and iso-osmotic solution (measurement solution containing 280 mM sorbitol) 30 min after removal of the 150 mM NaCl stress.

Fig. S4. Steady-state ﬂux of K^+^ in different root regions of 2*x* and 6*x I. trifida* under control conditions.

Fig. S5. Effects of OH· (1 mM Cu/A) on the transient net Ca^2+^ flux kinetics at the meristem, elongation, and mature root zones in 2*x* and 6*x I. trifida*.

Fig. S6. Effects of DPI on the NaCl-triggered Ca^2+^ influx at different root zones of 6*x I. trifida*.

Fig. S7. Effects of amiloride on the NaCl-triggered Na^+^ efflux in the root tissues of 6*x I. trifida*.

Fig. S8. Effects of EGTA and DPI on the NaCl-triggered Na^+^ efflux and intracellular Na^+^ accumulation in the root tissues of 6*x I. trifida*.

Fig. S9. Effects of NaCl stress (150 mM) on the transient net K^+^ flux kinetics at the meristem, elongation, and mature root zones in diploid (2*x*) and autotetraploid (4*x*) *A. thaliana* (Columbia).

Supplementary Figures S1-S9

## Author contributions

JS and ZYL designed the experiments; YL, YCY, JYS, and MYL performed the experiments; YL, TX, and JS analyzed the data; QHC, ZHT, and DFM provided the plant materials; and JS wrote the paper. All the authors read and approved the final manuscript.
