# Supplementary material for: Root-zone-specific sensitivity of K+-and Ca2+-permeable channels to H2O2 determines ion homeostasis in salinized diploid and hexaploid Ipomoea trifida
Source: J Exp Bot. 2019 Jan 25;70(4):1389–405. doi: 10.1093/jxb/ery461 (PMC6382330; doi:10.1093/jxb/ery461)
Supplement: Supplementary Figures S1-S9 [file ery461_suppl_supplementary-figures-s1-s9.pdf]

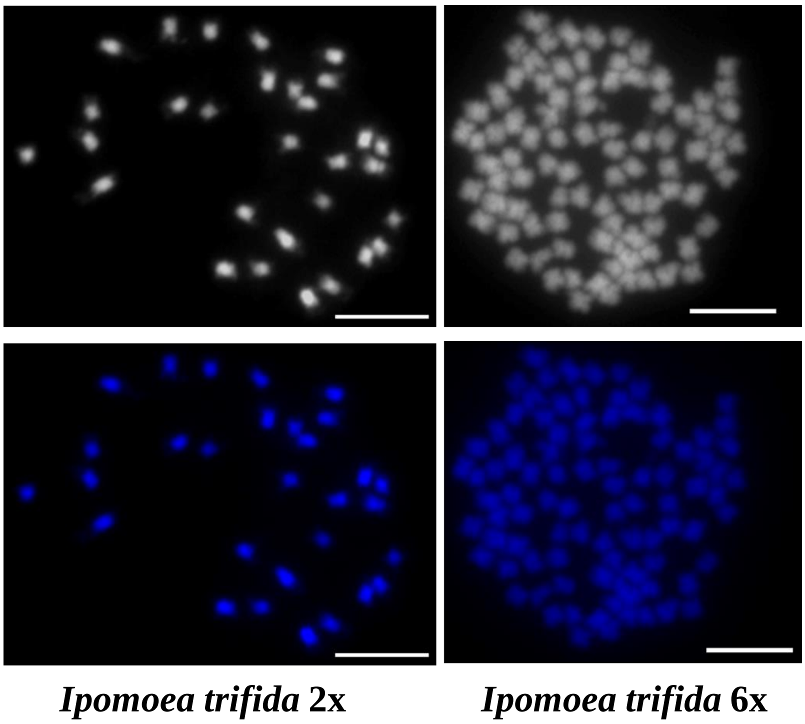

Fig. S1 Representative cytogenetic images show the mitotic metaphase of diploid (2x, 30 chromosomes) and hexaploid (6x, 90 chromosomes) *I. trifida*. Bar=5 μm.

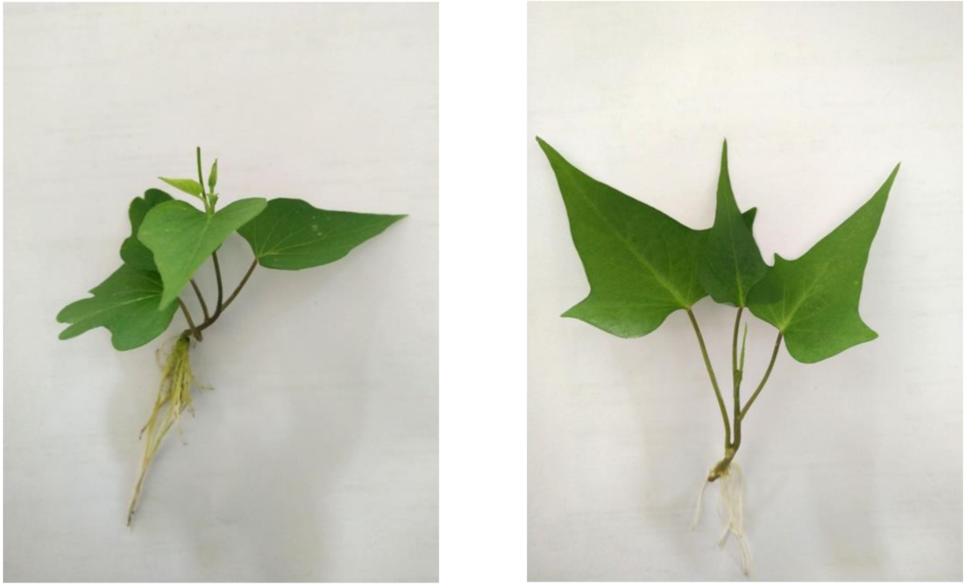

***I. trifida* (2x)** ***I. trifida* (6x)**

Fig. S2. Morphology of rooted seedlings of 2x and 6x *I. trifida*. Uniform seedlings were selected for experiments.

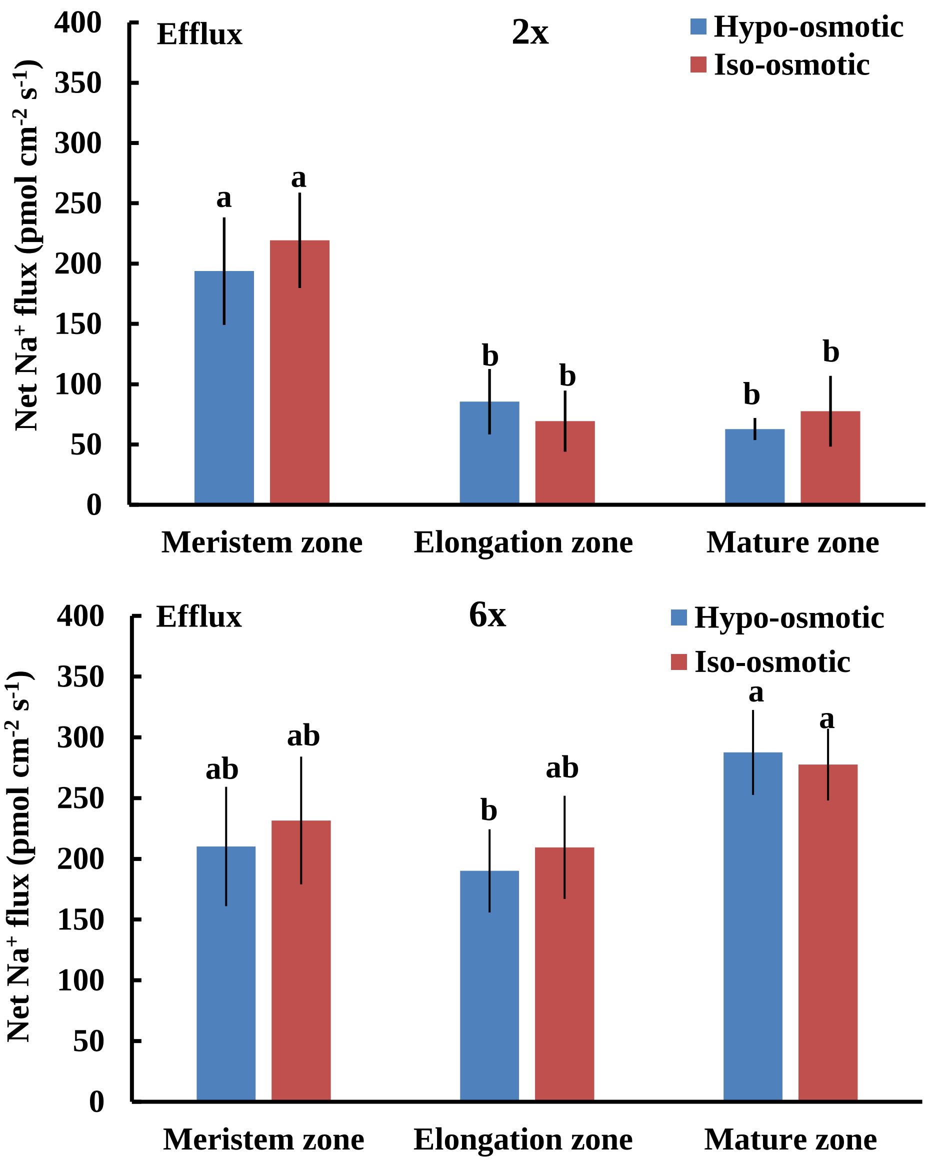

Fig. S3. Steady-state flux of Na^+ in the roots of 2x and 6x *I. trifida* measured in hypo-osmotic (measurement solution) and iso-osmotic solution (measurement solution containing 280 mM sorbitol) after 30 min of removal of the 150 mM NaCl stress. The steady-state Na^+ flux was measured from the meristem (300–600 μm from the tip), elongation (1–3 mm from the tip), and mature (10–15 mm from the tip) root zones after 24 h of NaCl treatment. Each column is equivalent to the mean of the 12 roots collected from 6 individual seedlings, and the bars represent the standard errors of the mean. Columns labeled with different letters indicate significant difference at $P < 0.05$.

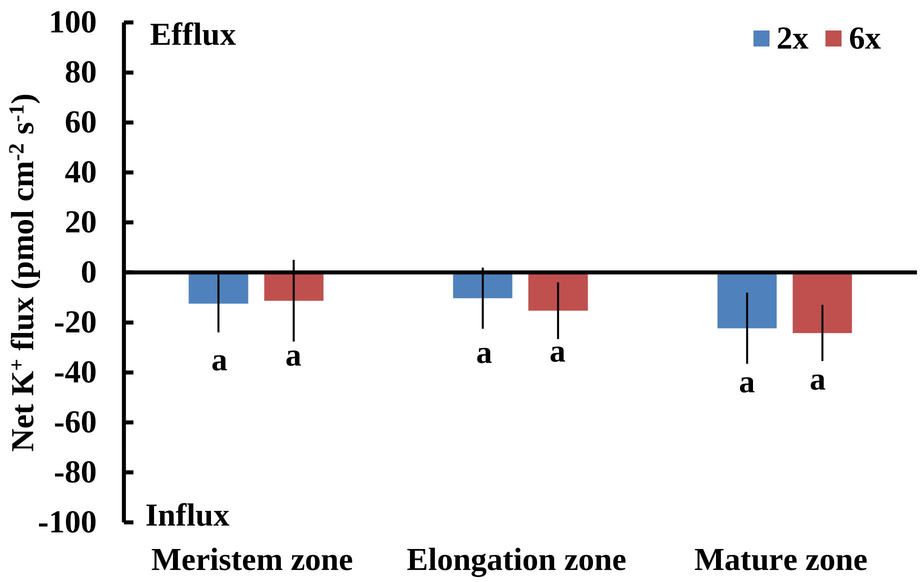

Fig. S4. Steady-state flux of K^+ in different root regions of 2x and 6x *I. trifida* under control condition. The steady-state K^+ flux was measured from the meristem (300–600 μm from the tip), elongation (1–3 mm from the tip), and mature (10–15 mm from the tip) root zones. Each column is equivalent to the mean of 6 roots collected from 3 individual seedlings, and the bars represent the standard errors of the mean. Columns labeled with different letters indicate significant difference at $P < 0.05$.

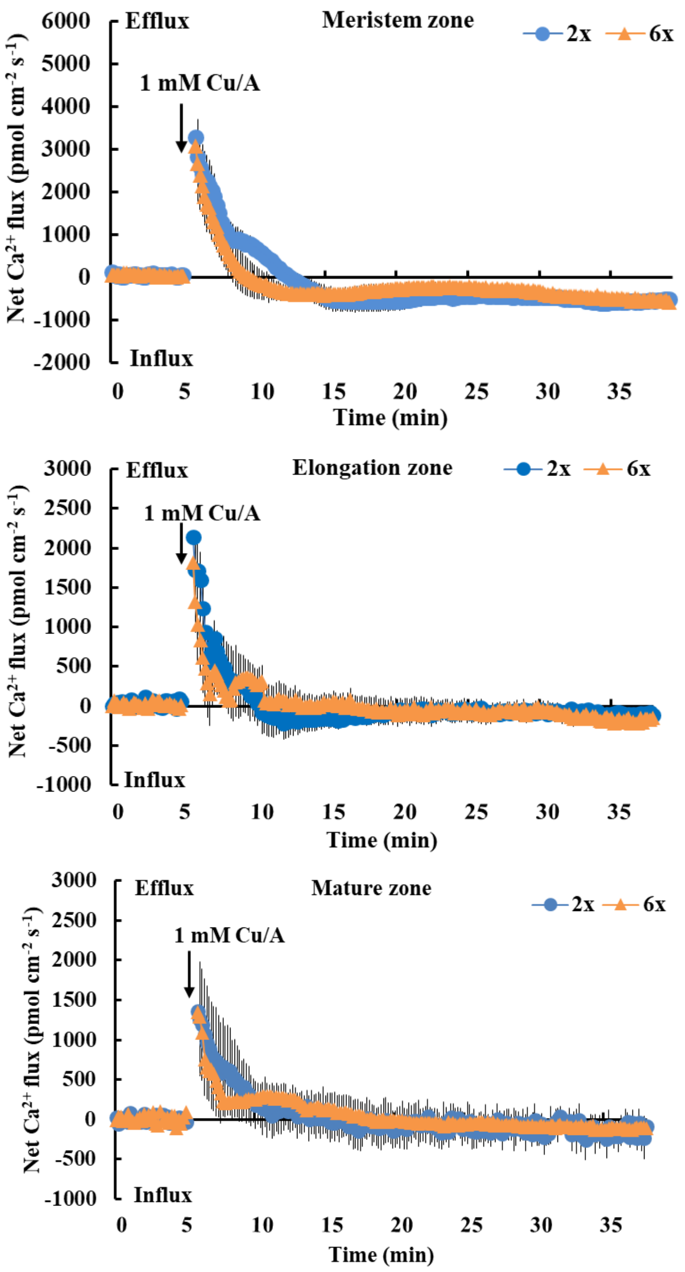

Fig. S5. Effects of OH· (1 mM Cu/A) on the transient net Ca^{2+} flux kinetics at the meristem (A: 500 µm from the tip), elongation (B: 3 mm from the tip), and mature (C: 15 mm from the tip) root zones in 2x and 6x *I. trifida*. Each point represents the mean of 12 roots collected from 6 individual plants.

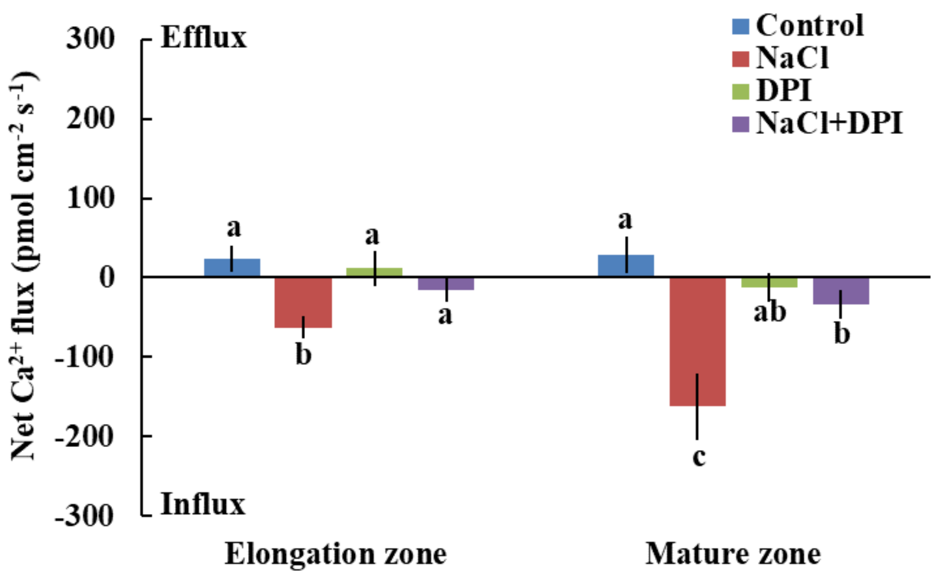

Fig. S6. Effects of DPI on the NaCl-triggered Ca^{2+} influx at different root zones of 6x *I. trifida*. The Ca^{2+} flux at the elongation (3 mm from the tip) and mature (15 mm from the tip) root zones was measured after 15 min of NaCl treatment in DPI-pretreated or non-pretreated roots. Columns show the mean rate of Ca^{2+} flux during measurements (approximately 10–12 min, 6 roots collected from 3 individual seedlings were measured) and the bars represent the standard errors of the mean. Different letters denote significant differences at $P < 0.05$.

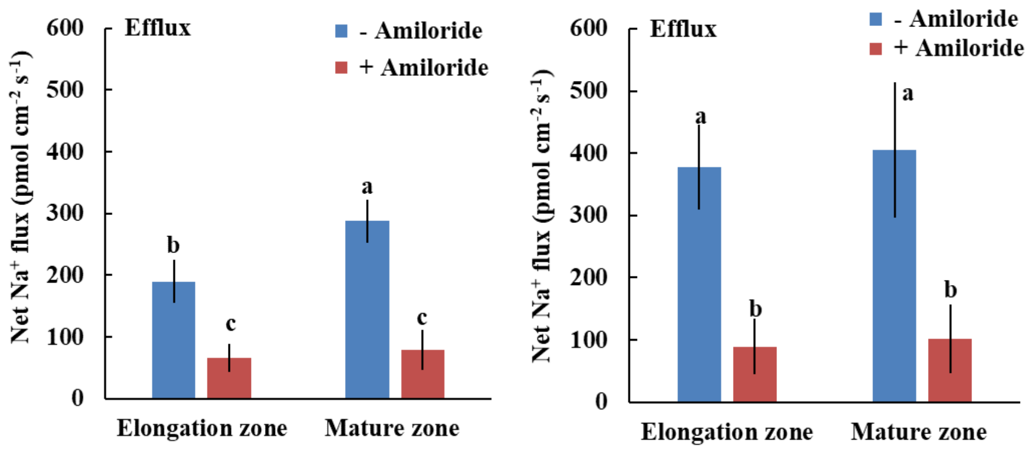

Fig. S7. Effects of amiloride on the NaCl-triggered Na^+ efflux in the root tissues of 6x *I. trifida*. The steady-state Na^+ flux at the elongation (1–3 mm from the tip) and mature (10–15 mm from the tip) root zones was measured after 24 h (left) and 5 day (right) of NaCl treatment in amiloride-treated or non-treated roots. Each column is equivalent to the mean of the 12 roots collected from 6 individual seedlings, and the bars represent the standard errors of the mean. Columns labeled with different letters indicate significant difference at $P < 0.05$.

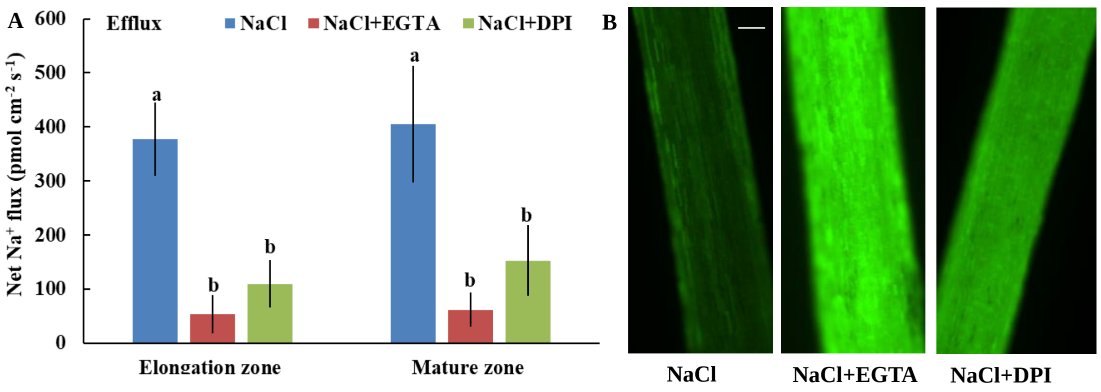

Fig. S8. Effects of EGTA and DPI on the NaCl-triggered Na^+ efflux and intracellular Na^+ accumulation in the root tissues of 6x *I. trifida*. (A) The steady-state Na^+ flux at the meristem (300–600 µm from the tip), elongation (1–3 mm from the tip) and mature (10–15 mm from the tip) root zones was measured after 5 days of NaCl treatment in EGTA/DPI-treated or non-treated roots. Each column is equivalent to the mean of the 12 roots collected from 6 individual seedlings, and the bars represent the standard errors of the mean. Columns labeled with different letters indicate significant difference at $P < 0.05$. (B) Intracellular Na^+ accumulation in the mature root zone as visualized by the CoroNa-Green fluorescent dye after 5 days of 150 mM NaCl treatment in the absence or presence of EGTA/DPI. One typical image (of 20) is shown. All images were taken using the same settings and exposure times to enable direct comparisons. Bars in C = 0.2 mm.

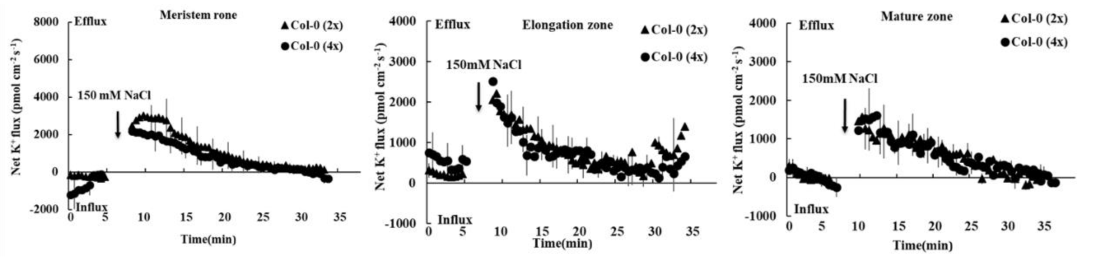

Fig. S9. Effects of NaCl stress (150 mM) on the transient net K^+ flux kinetics at the meristem (A: 300 µm from the tip), elongation (B: 2 mm from the tip), and mature (C: 10 mm from the tip) root zones in the diploid (2x) and autotetraploid (4x) *A. thaliana* (Columbia). Each point represents the mean of 6 individual plants (10-days-old).
